# The Anti-Vaccinators' Case in the Report of the Vaccination Commission

**Published:** 1897-03-06

**Authors:** 


### The Anti-Vaccinators' Case in the Report of the Vaccination Commission.

If out of the mouths of many witnesses truth may be expected, all who care to read should feel assured as to the truth about vaccination; for within the past fortnight we have been favoured with quite a series of papers dealing with the subject from almost every point of view.

Our readers will require no reminding of the findings of the Royal Commission on Vaccination; eleven out of the thirteen members of which, including a chairman so eminently fitted to form a critical judgment on evidence as Lord Herschell, the late Lord Chancellor, signed the report. This report is admirable in its moderately expressed, but none the less decisive, conclusions as to the value of vaccination. Asked how far vaccination protects against small-pox, these eleven commissioners say that it protects both against attack and death; that though the protection against attack afforded by infantile vaccination is most potent in the first nine or ten years, it is nevertheless considerable in later periods of life; that re-vaccination restores this protection; and that vaccination is beneficial according to the thoroughness with which it is performed. Asked as to the evil results of vaccination, their report holds that, considered in relation with the amount of vaccination performed, the dangers of the operation are insignificant, and such as they are, are diminishing. And, finally, asked whether means other than vaccination can be relied upon to prevent small-pox, such, for instance, as isolation hospitals, they tell us that they could not contemplate the consequences of trusting to these other means without dismay.

However great is the protection which vaccination affords, however, there is room for difference of opinion on the political question whether or no the State shall force unwilling parents thereto by fine and imprisonment. But few, if any, anti-vaccinators have confined themselves to this political question. They are pledged to the hilt that vaccination is not only a useless but a mischievous performance; and their case badly needed that some antidote should be found to the distasteful conclusions of this report. Accordingly, two members of the Commission who are to be regarded as friendly to the anti-vaccination cause, did their best to help anti-vaccinators out of their difficulty. After taking—by their own statement—a considerable share in the preparation of the Commissioners' report, Messrs. Collins and Picton withheld their signatures from this report, and published as an appendix a statement of dissent from the findings of their colleagues. This statement of dissent is, perhaps, the most ingenious and able defence of anti-vaccination that has ever yet been put forward.

All who have studied the statement of this minority of two must agree that the work has been ably done; indeed, in discussing many matters, Messrs. Collins and Picton have so well contrived that a careful reader, even if he is able to consult the huge folios of "Minutes of Evidence," will find no little difficulty in detecting if or when a fallacy exists. But it seems likely that these dissentients will now see some reason to regret their ingenuity. At the last meeting of the Epidemiological Society their statement was unkindly confronted by a paper read by Dr. J. C. McVail, who has made himself a master of vaccination literature, and above all of anti-vaccinators' methods. The result of his criticism is that there hardly remains a single material statement or argument adduced by the dissentients, which has not been controverted from the point of view of accuracy of fact or quotation, of sufficiency of reasoning, or of consistency with previous statements made by one or other of them. That Dr. McVail could point without difficulty to inaccuracies, and to fallacies of argument was perhaps to be expected. But the most damaging part of his indictment was the ample demonstration which he was able to give of what it is hardly too much to term the dishonest suggestiveness of the document—how, for instance, a quotation which with its context means one thing, had been pulled out of its place, so that the authority quoted is made to say a very different thing; how facts and figures have similarly been improperly wrenched from their true positions; in short, how the methods of the anti-vaccinator, so often exposed, have been again resorted to. It is not too much to say that Dr. McVail, using no other weapons than facts, for which he gives his authority, and a hard Scot's logic, has succeeded in utterly discrediting the statement of dissent put forward by these two Commissioners, and we confidently hope that the Epidemiological Society will take steps to secure a free circulation of this most valuable addition to the armament of those who maintain the utility of vaccination.

